# Feasibility of high-intensity interval training and moderate-intensity continuous training in adults with inactive or mildly active Crohn’s disease: study protocol for a randomised controlled trial

**DOI:** 10.1186/s40814-017-0133-z

**Published:** 2017-04-03

**Authors:** Garry A. Tew, Roger Carpenter, Michael Seed, Simon Anderson, Louise Langmead, Caroline Fairhurst, Lindsay Bottoms

**Affiliations:** 1 Department of Sport, Exercise and Rehabilitation, Northumbria University, Newcastle, NE1 8ST UK; 2 School of Health, Sport and Bioscience, University of East London, Stratford Campus, London, E15 4LZ UK; 3 Guy’s and St Thomas’ NHS Foundation Trust, London, UK; 4 Digestive Diseases Clinical Academic Unit, Barts and the London NHS Trust, London, UK; 5 York Trials Unit, University of York, Heslington, York YO10 5DD UK; 6 Centre for Psychology and Sports Science, Life and Medical Sciences, University of Hertfordshire, CP Snow Building, College Lane, Hatfield, AL10 9AB UK

**Keywords:** Crohn’s disease, Inflammatory bowel disease, Physical therapy, Exercise therapy, Rehabilitation, Randomised controlled trial, Feasibility studies

## Abstract

**Background:**

Structured exercise training has been proposed as a useful adjunctive therapy for Crohn’s disease by improving immune function and psychological health, reducing fatigue and promoting gains in muscle and bone strength. However, the evidence for exercise in Crohn’s disease is sparse, with only a handful of small prospective trials [1, 2], with methodological limitations, including the use of non-randomised and non-controlled study designs and small sample sizes. Here, we describe the protocol for a study that aims to assess the feasibility and acceptability of two common types of exercise training—high-intensity interval training (HIIT) and moderate-intensity continuous training (MICT)—in adults with inactive or mildly active Crohn’s disease (CD).

**Methods:**

This is a randomised, controlled, assessor-blinded, feasibility trial with three parallel groups. Forty-five adults with inactive or mildly active Crohn’s disease will be randomly assigned 1:1:1 to HIIT, MICT or usual care control. Participants in the HIIT and MICT groups will be invited to undertake three sessions of supervised exercise each week for 12 consecutive weeks. HIIT sessions will consist of ten 1-min intervals of cycling exercise at 90% of peak power output separated by 1 min of active recovery. MICT sessions will involve 30 min of continuous cycling at 35% of peak power output. Participants will be assessed before randomisation and 13 and 26 weeks after randomisation. Feasibility outcomes include rates of recruitment, retention and adherence. Interviews with participants will explore the acceptability of the exercise programmes and study procedures. Clinical/health outcomes include cardiorespiratory fitness, body mass index, resting blood pressure, markers of disease activity (faecal calprotectin and Crohn’s Disease Activity Index) and activated T cell cytokine profiles. Study questionnaires include the Inflammatory Bowel Disease Quality of Life Questionnaire, EQ-5D-5L, IBD Fatigue Scale, Hospital and Anxiety Depression Scale, and International Physical Activity Questionnaire.

**Discussion:**

This study will provide useful information on the feasibility and acceptability of supervised exercise training in adults with inactive and mildly active Crohn’s disease and will inform the design of a subsequent, adequately powered, multi-centre trial.

**Trial Registration:**

The trial is registered with the International Standard Randomised Controlled Trial Register (ISRCTN13021107). Date registration assigned was 02/12/2015.

## Background

Crohn’s disease (CD) is a relapsing inflammatory disease, affecting the gastrointestinal tract, which often presents with abdominal pain, fever, bowel obstruction or diarrhoea. In 2012, there were reported to be at least 115,000 people in the UK with CD [3]. For people in remission or with less active disease, treatment is largely directed at preventing relapse and maintaining or improving health-related quality of life [4].

Management options for CD include drug and/or biologic therapy, nutrition and diet modification, surgery and smoking cessation [4]. Physical exercise, whilst not currently part of the recommended treatment pathway, has been proposed as a potentially useful adjunctive therapy for CD by improving immunological responses and psychological health, reducing fatigue and promoting gains in muscle and bone strength [3]. However, the evidence base for exercise in CD is sparse, with only a handful of relevant studies, many of which have methodological limitations, including the use of non-randomised and non-controlled study designs and small sample sizes [3].

The effects of exercise training depend largely on the type, intensity and volume of the exercise stimulus [5]. The most common model of exercise prescription in other chronic diseases has been moderate-intensity continuous training (MICT, e.g. 30 min of moderate-intensity continuous endurance-type exercise such as swimming, cycling or jogging thrice weekly). This is well tolerated and has beneficial effects on many different aspects of physical and mental health [6]. However, a growing body of evidence indicates that shorter bouts of intense intermittent exercise—so-called ‘high-intensity interval training’ (HIIT, e.g. 1 min of hard exercise followed by 1 min of easy exercise repeated ten times)—can elicit similar physiological adaptations compared to more lengthy continuous exercise [7]. There has only been one published report investigating HIIT in people with CD to date [8], which demonstrated a single session of cycle-based HIIT was well tolerated and did not significantly increase inflammatory biomarkers in a group of 15 teenage patients. A greater understanding of the acceptability of, and chronic adaptations to, different types of exercise training is clearly warranted for safe and effective exercise promotion strategies in adults with CD.

We hypothesise that supervised exercise training will be an effective means of improving health-related quality of life, fatigue and physical and mental health in people with inactive or mildly active CD. Before embarking on an adequately powered randomised controlled trial to test this hypothesis, we have planned a feasibility study that will address several areas of uncertainty. For example, fatigue and a perceived inability to manage exercise have been cited as barriers to exercise in people with inflammatory bowel disease [9]. This raises questions about how easy it will be to recruit and retain participants in a supervised exercise training trial. The objectives of the present feasibility study are the following:

1. To explore the acceptability, safety and potential benefits of HIIT and MICT in people with inactive and mildly active CD, relative to usual care.
2. To evaluate the feasibility of conducting a future large-scale randomised controlled trial to assess the clinical effectiveness of exercise training in people with CD in terms of recruitment, retention, adherence and acceptability.
3. To identify a primary outcome and estimate the parameters required to inform sample size calculation for an adequately powered randomised controlled trial.

## Methods

### Study design

EXACT (Exercise for Adults with Crohn’s disease Trial) is a multi-centre, three-arm, parallel-group, randomised controlled feasibility trial. Following baseline assessment, participants will be randomly assigned 1:1:1 to usual care plus HIIT, usual care plus MICT or usual care alone. Study assessments will be conducted at baseline and at 13 and 26 weeks after randomisation.

### Study setting

Participants will be recruited from three National Health Service (NHS) Hospital Trusts: Guy’s and St Thomas’ NHS Foundation Trust, Barts Health NHS Trust and Hampshire Hospitals NHS Foundation Trust. The exercise programmes will be delivered in the exercise science facilities of the University of East London and the University of Winchester. The study is sponsored by the University of Hertfordshire.

### Eligibility criteria

#### Inclusion criteria

- Age 16–65 years.
- Clinical diagnosis of CD for at least 4 weeks before the screening visit.
- Inactive (<150 on Crohn’s Disease Activity Index [CDAI]) or mildly active (150–219 on CDAI) CD assessed no greater than 4 weeks before the screening visit.
- Faecal calprotectin <250 mcg/g recorded no greater than 4 weeks before the screening visit.
- Stable medication for at least 4 weeks before the screening visit.
- Willing and able to provide written informed consent and complete the study questionnaires.
- Willing and able to travel to the research centre for assessment visits and exercise sessions.

#### Exclusion criteria

- Absolute contraindications to exercise testing and training as defined by the American College of Sports Medicine [10].
- Coexistent serious autoimmune disease such as rheumatoid arthritis or systemic sclerosis.
- Planned major surgery within the first 3 months after randomisation.
- Pregnant
- Female planning pregnancy within the first 3 months after randomisation.
- Poor tolerability of venepuncture.
- Lack of adequate venous access for required blood sampling.
- Current participation in >90 min/week of purposeful exercise, such as jogging or swimming.
- Participation in another clinical trial for with concurrent participation is deemed inappropriate.

### Interventions

#### High-intensity interval training (HIIT)

Participants allocated to the HIIT group will be invited to complete three sessions of HIIT each week for 12 consecutive weeks. It is anticipated that most sessions will be completed within normal office hours (i.e. 0900 to 1700) on Monday, Wednesday and Friday each week; however, early morning, evening and weekend sessions will be offered to facilitate attendance of participants who cannot attend on these days or times. The exercise will be performed on a calibrated, stationary upright cycle ergometer. Each session will begin with 5 min of cycling at 15% of the peak power output (*W*peak) recorded during the baseline cardiopulmonary exercise test. The main body of each session will involve ten 1-min bouts of cycling at 90% of *W*peak, interspersed with 1-min bouts of cycling at 15% of *W*peak [11]. The session will end with 3 min of cycling at 15% of *W*peak. Similar protocols of HIIT have been shown to be safe and effective for improving cardiorespiratory fitness in various clinical and non-clinical populations [12, 13]. Incremental cycle exercise testing, without the analysis of expired gases and blood lactate responses, will be performed during the final sessions of the fourth and eighth week of training (i.e. at sessions 12 and 24 of 36) and will replace the normal exercise training of those sessions. The peak power output recorded during these tests will be used to adjust the power output used within the training sessions (i.e. the training will be progressed after 4 and 8 weeks according to changes in peak power output assessed using incremental cycle exercise testing).

Each session will be supervised by a research assistant who will have received specific training from the investigators in how to deliver the exercise protocol. A maximum of four participants will be trained per session. A specifically designed exercise session case report form (CRF) will be completed for each exercise session. Prior to exercise, and 10-min post-exercise, the participant’s resting heart rate and blood pressure will be recorded (Omron, M2, The Netherlands). Differential ratings for breathlessness and leg exertion will be assessed using Borg’s CR-10 scale [14] before exercise, immediately post, and 10-min post-exercise. In addition, participants will be asked to rate their breathlessness and leg exertion at 2.5, 7.5, 42.5, 47.5, 92.5 and 97.5% of exercise completed. These time points were chosen to incorporate both interval and recovery periods during the HIIT protocol [15]. Participants’ heart rate will also be recorded using Polar heart rate monitors at 2.5, 42.5 and 92.5% of exercise completed. The one item Feeling Scale (FS; [16]) will be used to measure general affective valence (i.e. pleasure and displeasure). Participants will be prompted at the beginning of each exercise visit with the following instructions: ‘While participating in exercise, it is common to experience changes in mood. Some individuals find exercise pleasurable, whereas others find it to be unpleasant. Additionally, feeling may fluctuate across time. That is, one might feel good and bad a number of times during exercise. When asked, please tell me how you feel at that current moment using the scale below.’ The feeling scale is scored on an 11-point bipolar scale ranging from −5 (very bad) to +5 (very good). The FS will be administered pre-, immediately post and 10-minutes post-exercise. To assess in-task affect, the FS will be administered at 2.5, 42.5 and 92.5% of exercise completed. The collection of such data will permit a detailed quantification of the exercise intervention. The research assistant will be required to email a copy of the anonymised exercise session CRF for sessions 1, 13 and 25 to a member of the trial management team so that they can confirm that the CRF and sessions are being completed appropriately.

Overall enjoyment of the exercise programme will be assessed during the week 13 assessment, using the validated multi-dimensional Physical Activity Enjoyment Scale (PACES) [17]. This will typically be 3–7 days following completion of the overall programme, allowing participants’ reflection and evaluation time. Participants in the control group will not be required to complete this.

#### Moderate-intensity continuous training (MICT)

Participants allocated to the MICT group will be invited to complete three cycle-based exercise sessions each week for 12 weeks, with each session involving a warm-up and cool-down of cycling at 15% of *W*peak. The main body of each exercise session in this group involves 30 min cycling at 35% of *W*peak. This programme has been selected because it has been shown to elicit a similar energy expenditure compared with the HIIT programme [11]. As described for the HIIT group above, the training will be progressed according to changes in peak power output re-assessed after 4 and 8 weeks. Heart rate, perceived exertion and general affective valence will be recorded at the same relative time points as in the HIIT group.

Both exercise programmes will be delivered in addition to usual care. Participants in both exercise groups will be able to claim up to £10.90 per session for travel expenses.

After completing the 12-week intervention period, all exercise group participants will receive an individualised exercise programme that they can manage on their own. The exercise prescription will largely reflect the content of the supervised exercise programme; however, for example, the mode of exercise may change if a cycle ergometer cannot be easily accessed by the participant.

#### Usual care control group

This group will not receive any supervised exercise or be given any specific recommendations regarding exercise. However, once control participants have completed the 26-week assessment, they will be offered a one-to-one exercise consultation via telephone with the research assistant in which they will discuss the benefits of exercise training, barriers and facilitators of exercise, exercise guidelines and personal goals and action plans. The aim of offering this consultation to control participants is to help minimise the potential for resentful demoralisation that may occur through being allocated to this group [18].

### Outcome measures

#### Feasibility and acceptability outcomes

The main focus of this study is feasibility and acceptability of procedures for recruitment, allocation, measurement, retention and the intervention procedures. Recruitment rates will be measured as rate of invited participants who are eligible and consenting and will be reported in a Consolidated Standards of Reporting Trials (CONSORT) participant flowchart. Acceptability of allocation procedures will be assessed by examining reasons for dropout in discontinuing participants and comparing attrition rates between the three study groups and between participants who did and did not receive their preferred allocation (assessed prior to randomisation). Suitability of measurement procedures will be evaluated based on completion rates and rates of missing data. Attrition rates will be established as discontinuation of intervention and loss to follow-up measurement for all groups. The acceptability of the exercise programmes will be assessed using session adherence rates, measures of exercise enjoyment (see the Interventions section) and participant and provider feedback via telephone interviews conducted after the 26-week follow-up visit. The participant interviews will last up to 30 min and will cover perceived benefits and negative consequences from participating in the study, feedback regarding specific design features of the study (including the exercise protocol and assessment procedures) and perceptions of barriers and facilitators to intervention participation. The safety of exercise training will also be assessed by exploring rates of disease relapse at 13 weeks, reasons for dropout from the exercise programmes and the number and type of adverse events, both CD specific and general, which occur in each group.

#### Behavioural and health-related outcomes

In addition to acceptability and feasibility, we will assess the following outcome measures in all participants before randomisation and 13 weeks after randomisation: body mass, stature, waist circumference, blood pressure, resting heart rate, cardiorespiratory fitness (ventilatory threshold and peak oxygen uptake), disease status and relapse (CDAI), intestinal inflammation (faecal calprotectin) and blood markers of inflammation* (T lymphocyte subsets [Th1/Th2/Th17] and various cytokines including IL-6, IL-10, TNF-α and C-reactive protein [via 2 × 1-mL blood samples drawn from non-fasted participants at each time point]). Relapse will be defined as an increase in CDAI score of at least 100 points, to a level greater than 150 [19]. Questionnaires will also be administered before and 13 and 26 weeks after randomisation, including the Inflammatory Bowel Disease Quality of Life Questionnaire (IBDQ) [20], EuroQol EQ-5D-5L [21], IBD Fatigue Scale [22], Hospital and Anxiety Depression Scale (HADS) [23] and International Physical Activity Questionnaire (IPAQ)—Short Form [24].

*NOTE: The blood markers of inflammation will also be assessed after 6 weeks of exercise training in the participants in the *two exercise groups only*. Here, venous blood will be collected at the start of the first session of the 7th week of exercise training (i.e. before commencing the 19th exercise session). This additional blood assessment has been included to provide an indication of the time course of changes in circulating inflammatory markers with chronic exercise training.

The assessment of cardiorespiratory fitness will be achieved by measuring ventilatory threshold and peak oxygen uptake. Participants will undergo cardiopulmonary exercise testing using an incremental protocol on an electronically braked cycle ergometer (Lode, Corival, The Netherlands). After 2 min of unloaded cycling, the intensity of exercise will be increased by 15–25 W min^−1^. The target cadence will be 60–80 rev min^−1^. Participants will be encouraged to continue cycling to volitional exhaustion. Heart rate monitoring will be performed continuously (Polar, S610i, Finland). Rating of perceived exertion (RPE; Borg 6–20 scale [14]) will be recorded at the end of every minute. The volume of oxygen consumed during exercise was calculated from minute ventilation and measured using a pneumotachometer and simultaneous breath-by-breath analysis of expired gas fractions (Cosmed, K5, Italy). Gas analysers and flow probes will be calibrated before each test. Oxygen consumption will be expressed relative to body mass (mL∙kg^−1^∙min^−1^). Ventilatory threshold will be determined by an independent exercise physiologist blinded to group allocation using the v-slope and ventilatory equivalents methods [25]. Peak oxygen uptake will be calculated as the highest consecutive 20-s period of oxygen uptake data in the last minute before volitional exhaustion. For an exercise test to be considered valid, at least two of the following criteria must be satisfied: respiratory exchange ratio ≥1.1, RPE ≥17, heart rate ≥95% age-predicted maximum (220 minus age), post-exercise blood lactate ≥6.0 mM.

### Participant timeline

**Fig. 1:**
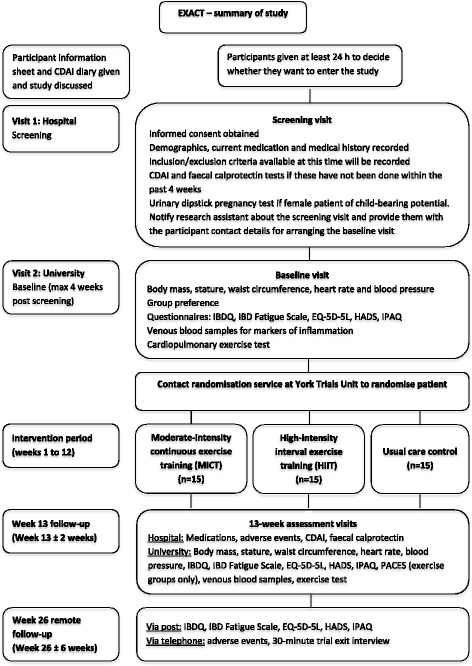
Schematic diagram of recruitment and assessment

Figure 1 illustrates the process of enrolling participants into the study, the interventions being compared, and timing of assessments and hospital visits for the participants in the trial.

### Sample size

As this is a feasibility study, no formal sample size calculation was performed [26]. Instead, this study follows sample size recommendations for pilot randomised controlled trials [27] and aims to have at least 12 participants per study arm who provide full data. We aim to recruit 15 participants to each group (i.e. total sample size of 45) to allow up to 20% attrition, which is appropriate based on our exercise studies in other clinical populations [28–30]. This number of participants is deemed adequate to provide sufficient information on key feasibility issues such as recruitment, retention and acceptability of the exercise programmes.

### Recruitment

Recruitment methods will include advertisements through the local media and charity groups (e.g. Crohn’s and Colitis UK), invitations to participants in previous studies who have given their consent to be contacted regarding future research projects, and liaisons with direct care teams at recruiting NHS Hospital Trusts. For the latter of these methods, a member of the direct care team will identify potentially eligible patients via review of medical notes ahead of clinics and database screening of clinic letters.

Potentially eligible patients will be screened for participation in the study by a member of the direct care team with eligibility confirmed by a delegated clinical co-investigator. Data used for calculating the CDAI and faecal calprotectin results must have been collected no greater than 4 weeks before the screening visit. Once an eligible patient has consented to study participation (consent being obtained in writing by a research nurse), their contact details will be forwarded to the research assistant, who will contact the participant to book them in for their baseline assessment, which must be performed within 4 weeks from when the screening assessments have been fully completed.

### Randomisation and allocation concealment

Following collection of all baseline measurements, participants will be randomly assigned 1:1:1 to HIIT, MICT or usual care control. Randomisation will be achieved using a computer-generated randomisation schedule stratified by centre and baseline disease status (inactive [CDAI <150] vs. mildly active [CDAI 150–219]) using permuted blocks of random sizes. The block sizes and allocation sequence will not be disclosed to ensure concealment. A statistician at York Trials Unit will manage the randomisation process. The intervention facilitator (research assistant) will request email notification of a participant’s treatment allocation after baseline assessments have been completed. They will then inform the participant of their allocation (i.e. participants will not be blinded to the treatment allocation).

### Blinding

Blinding of trial participants and the intervention facilitator is not possible. Questionnaires will be completed by the participants independently and checked by a researcher for completeness. Anthropometric, cardiorespiratory fitness and disease activity outcomes will be assessed by researchers blinded to group allocation. Participants will be asked not to disclose their allocation to the technician.

### Statistical analysis

A detailed analysis plan will be prepared and agreed with the Trial Management Group and Trial Steering Committee, before all of the data has been collected. Analyses will be conducted in STATA (StataCorp, 2013) using the principles of intention-to-treat. The flow of participants through each stage of the trial will be presented in a CONSORT diagram. Descriptive statistics will be used to characterise the groups at baseline and to present the feasibility outcomes. Although determining differences in clinical outcomes between the three arms is not the primary purpose of this trial, comparisons will be undertaken to investigate the feasibility of studying these outcomes and to calculate estimates for the likely effect sizes and 95% confidence intervals. As recommended in guidelines for good practice for the analysis of pilot studies [23], the focus of the results will be on the estimates of the treatment effects rather than statistical significance and as such no hypothesis testing will be undertaken. Differences between the two comparison groups will be presented in the form of an unadjusted mean difference for continuous outcomes, and an odds ratio for binary outcomes, with their associated 95% confidence intervals.

### Criteria for success

This feasibility trial will be deemed successful and lead to the development of a proposal for an adequately powered randomised controlled trial if:

- 1. At least one of the exercise programmes is shown to be acceptable, based on a combination of data from participant interviews, the number and type of adverse events in each group and exercise session adherence rates (at least 67% of participants completing at least 24 of the 36 sessions).
- 2. At least 24 patients are recruited during the 12-month recruitment window (based on recruitment from three NHS Hospital Trusts and 1–2 recruits per site per two months).
- 3. At least 67% of participants provide valid cardiorespiratory fitness, disease activity (CDAI) and quality of life (IBDQ) data at the 13-week follow-up visit.

The criteria for success will provide the basis for interpreting the results of this study and determine whether it is appropriate to proceed to a full trial and if any modifications need to occur for a full trial to be feasible.

### Ethical and safety considerations

The research study will follow the Health Research Authority’s processes for gaining NHS permissions and approvals. We are confident that the assessment procedures and exercise programmes will be safe. Exercise testing has been demonstrated to be safe for patients with CD [30] and other chronic diseases [31, 32]. Nevertheless, patients’ suitability for the study will be assessed by a clinical co-investigator, and participants will be closely monitored during all training and testing sessions. Furthermore, exercise sessions will be supervised by experienced staff who are trained in resuscitation. Exercise training will be discontinued if a clinical co-investigator deems it necessary (e.g. if a patient experiences a disease relapse) or if the participant withdraws consent. The trial will also be subject to oversight from an independent Trial Steering Committee.

### Safety reporting

Adverse event reporting will be conducted in accordance with the Sponsor’s Adverse Event Reporting Procedures. A clinical co-investigator will be responsible for determining the causality and seriousness of adverse events and ensuring that appropriate action is taken. Information about adverse events will be collected from the beginning of any study-related procedure (i.e. when written consent has been obtained). The adverse event reporting period will stop at the participant’s final trial contact, i.e. at the 26-week follow-up.

We will record all serious adverse events, as well as all non-serious adverse events that are either deemed to be related to participation in the research or result in withdrawal from an exercise programme or the study. Serious adverse events are defined as any untoward medical occurrence that falls in one of the following criteria: results in death; is life threatening; requires unplanned or prolonged hospitalisation; results in persistent or significant disability or incapacity; or results in a congenital abnormality or birth defect. Non-serious events are defined as untoward medical occurrences that do not fulfil any of the serious adverse event criteria.

### Data management

Paper case report forms will be used to collect study outcome data. An exercise session case report form will also be completed for each exercise session for participants in the two exercise intervention groups. Examples of the case report forms will be stored in the Trial Master File. Each centre’s personnel will receive training from members of the Trial Management Group in the study requirements, including how to complete the case report forms, how to conduct the assessments (e.g. cardiopulmonary exercise tests, blood sampling) and how to deliver the interventions. The study case report forms will be stored in participant files, which in turn will be stored in numerical order in a secure, restricted access location. The Chief Investigator (LB) and Trial Manager (GT) will regularly review case report forms to ensure that they are being completed appropriately, and they will conduct a site monitoring visit once a site has recruited three participants.

Data on paper case report forms will be manually entered into customised spreadsheets in Microsoft Excel. Entered data will be double-checked by a second researcher. Once completed, these spreadsheets will be password encrypted and then transferred electronically to the statistician at York Trials Unit using the University of York’s secure document DropOff Service. Data validation plans will be implemented by the statistician to identify data points that do not fall within an expected range of values. Following data cleaning, the data will be summarised and analysed according to the statistical analysis plan.

Essential trial documentation (i.e. the documents which individually and collectively permit evaluation of the conduct of a clinical trial and the quality of the data produced) will be kept with the Trial Master File and Investigator Site Files. The Sponsor will ensure that this documentation will be retained for a minimum of 5 years after the conclusion of the trial to comply with standards of Good Clinical Practice. Case report forms will be stored for a minimum of 5 years after the conclusion of the trial as paper records and a minimum of 20 years in electronic format. All paper records will be stored in a secure storage facility or off-site by the University of Hertfordshire. All electronic records will be stored on a password-protected server.

### Dissemination

The dissemination strategy for this research will be to inform a wide range of local, national and international audiences about the results and conclusions. It must, however, be remembered as part of this strategy that the current project is preliminary work aimed at informing a subsequent definitive clinical trial.

Health professionals—we aim to publish our research in journals that cover the relevant medical specialties and with preference for those that deposit publications in open access databases to increase free dissemination. In addition, we aim to present this research at appropriate national and international conferences.

Users—from this perspective, we aim in the first instance to collaborate with our patient representatives and local experts in the patient and public involvement to best facilitate user dissemination. We plan to write a specific news piece that will be forwarded to appropriate groups and organisations.

Service managers—as an exploratory study, it is unlikely that results from this study will directly influence commissioning processes in the short term. Moreover, we will engage with appropriate primary and secondary care groups to discuss support for our proposed definitive study leading on from this research.

## Discussion

Regular physical exercise may benefit several aspects of physical and mental health in people with inflammatory bowel disease; however, the evidence base is sparse. The EXACT study is a rigorously designed randomised controlled feasibility trial that will provide useful preliminary information about the acceptability and effectiveness of two common modes of supervised exercise training (high-intensity interval training and moderate-intensity continuous training) in people with inactive or mildly active Crohn’s disease. The findings will inform the development of a future adequately powered, multi-centre, randomised controlled trial.

### Trial status

Recruitment started in April 2016 and is ongoing.

## Abbreviations

CD: Crohn’s disease
CDAI: Crohn’s Disease Activity Index
CRF: Case report form
EXACT: Exercise for Adults with Crohn’s disease Trial
FS: Feeling Scale
HADS: Hospital Anxiety and Depression Scale
HIIT: High-intensity interval training
IBD: Inflammatory bowel disease
IBDQ: Inflammatory Bowel Disease Questionnaire
IPAQ: International Physical Activity Questionnaire
MICT: Moderate-intensity continuous training
NHS: National Health Service
PACES: Physical Activity Enjoyment Scale
RPE: Rating of perceived exertion
*W*peak: Peak power output
